# Antibacterial activity of ethoxzolamide against *Helicobacter pylori* strains SS1 and 26695

**DOI:** 10.1186/s13099-020-00358-5

**Published:** 2020-04-15

**Authors:** Mohammad M. Rahman, Alexandra Tikhomirova, Joyanta K. Modak, Melanie L. Hutton, Claudiu T. Supuran, Anna Roujeinikova

**Affiliations:** 1 Department of Microbiology, Monash University, Clayton, VIC 3800 Australia; 2 Infection and Immunity Program, Monash Biomedicine Discovery Institute, Monash University, Clayton, VIC 3800 Australia; 3 Neurofarba Department, Sezione di Scienze Farmaceutiche, Universita degli Studi di Firenze, Via U. Schiff 6, Sesto Fiorentino, 50019 Florence, Italy; 4 Department of Biochemistry and Molecular Biology, Monash University, Clayton, VIC Australia

**Keywords:** Mutation frequency, Ethoxzolamide, Genome sequencing, Resistance

## Abstract

With the rise of bacterial resistance to conventional antibiotics, re-purposing of Food and Drug Administration (FDA) approved drugs currently used to treat non-bacteria related diseases as new leads for antibacterial drug discovery has become an attractive alternative. Ethoxzolamide (EZA), an FDA-approved diuretic acting as a human carbonic anhydrase inhibitor, is known to kill the gastric pathogenic bacterium *Helicobacter pylori* in vitro via an, as yet, unknown mechanism. To date, EZA activity and resistance have been investigated for only one *H. pylori* strain, P12. We have now performed a susceptibility and resistance study with *H. pylori* strains SS1 and 26695. Mutants resistant to EZA were isolated, characterized and their genomes sequenced. Resistance-conferring mutations were confirmed by backcrossing the mutations into the parent strain. As with P12, resistance to EZA in strains SS1 and 26695 does not develop easily, since the rate of spontaneous resistance acquisition was less than 10^−8^. Acquisition of resistance was associated with mutations in 3 genes in strain SS1, and in 6 different genes in strain 26695, indicating that EZA targets multiple systems. All resistant isolates had mutations affecting cell wall synthesis and control of gene expression. EZA’s potential for treating duodenal ulcers has already been demonstrated. Our findings suggest that EZA may be developed into a novel anti-*H. pylori* drug.

## Background

*Helicobacter pylori* is a significant human pathogen causing gastric and duodenal ulcers which, if left untreated, can lead to cancers such as mucosa-associated lymphoid tissue lymphoma and gastric adenocarcinoma [1, 2]. With the rise of antibiotic resistance, the efficacy of existing treatment regimes has gradually decreased over the years [3–5], and the World Health Organization (WHO) recognized the urgent need for novel anti-*H. pylori* therapies in 2017 by listing clarithromycin-resistant *H. pylori* among other high priority pathogens for antimicrobial research development [6].

Owing to the spread of bacterial resistance to conventional antibiotics, re-purposing of Food and Drug Administration (FDA) approved drugs currently used to treat non-bacteria related diseases as new leads for antibacterial drug discovery has been recognized as an attractive alternative. Ethoxzolamide (EZA)—known under the name Cardrase—is an FDA-approved anti-glaucoma drug that acts by inhibiting the human metalloenzyme carbonic anhydrase (hCA) which catalyzes the interconversion of CO_2_ and bicarbonate [7, 8]. EZA has come to attention in the *H. pylori* field of research due to its unexpected ‘side effect’ of healing stomach ulcers. In a pilot study, administration of EZA resulted in ulcer healing in 98% of the patients [9]. The additional fact that 2 years following treatment, the ulcer recurrence rate in the tested subjects (11% [9]) was significantly lower than that typically observed with classical antacid drugs (34–79%), and close to that achieved by the antibiotic-based *H. pylori* eradication therapy [10], strongly suggested that EZA treatment cleared the *H. pylori* infection which caused ulcer disease in the first place.

Indeed, EZA has been shown to kill *H. pylori* P12 in vitro [11]. In addition, it was shown to be effective against clinical isolates resistant to conventional antibiotics, suggesting that EZA kills *H. pylori* via mechanisms different from those of metronidazole, clarithromycin, and amoxicillin. Developed as an inhibitor of hCAs, EZA is also known to inhibit activity of *H. pylori* α- and β-carbonic anhydrases (HpαCA and HpβCA) [12, 13]. The crystal structures of HpαCA bound to EZA and related sulfonamides demonstrated that these compounds act as competitive inhibitors mimicking the transition state of the reaction, and that their structure correlates well with their in vitro inhibitory properties [14, 15]. These observations suggest that the EZA inhibition of activity of *H. pylori* carbonic anhydrases likely plays an important role in the mechanism of its antibacterial action, although no spontaneous HpαCA and HpβCA mutants with resistance to EZA have been isolated so far. Furthermore, previous studies showed that in *H. pylori* strain P12, spontaneous resistance to EZA does not emerge easily [11], suggesting that it may be considered a lead compound for development of an anti-*H. pylori* drug with a novel mechanism of action. This work emphasized the necessity for further studies of EZA activity against different *H. pylori* strains.

Here, we present the results of a susceptibility and resistance study with *H. pylori* strains SS1 (mouse-adapted Sydney strain 1) and 26695. We show that spontaneous *H. pylori* strain SS1 and strain 26695 mutants resistant to EZA arise at a relatively low frequency (less than 10^−8^) and identify the genetic determinants that confer resistance. Our data indicates that acquisition of resistance leads to a complex genotype, likely the result of mechanisms involving systems other than α- and β-carbonic anhydrases themselves. Analysis of the commonalities between the resistance determinants in different *H. pylori* strains and implications for the understanding of the mechanism of anti-*H. pylori* activity of EZA are discussed.

## Results

### Antimicrobial activity of EZA against H. pylori strains SS1 and 26695 is time- and concentration-dependent

**Fig. 1:**
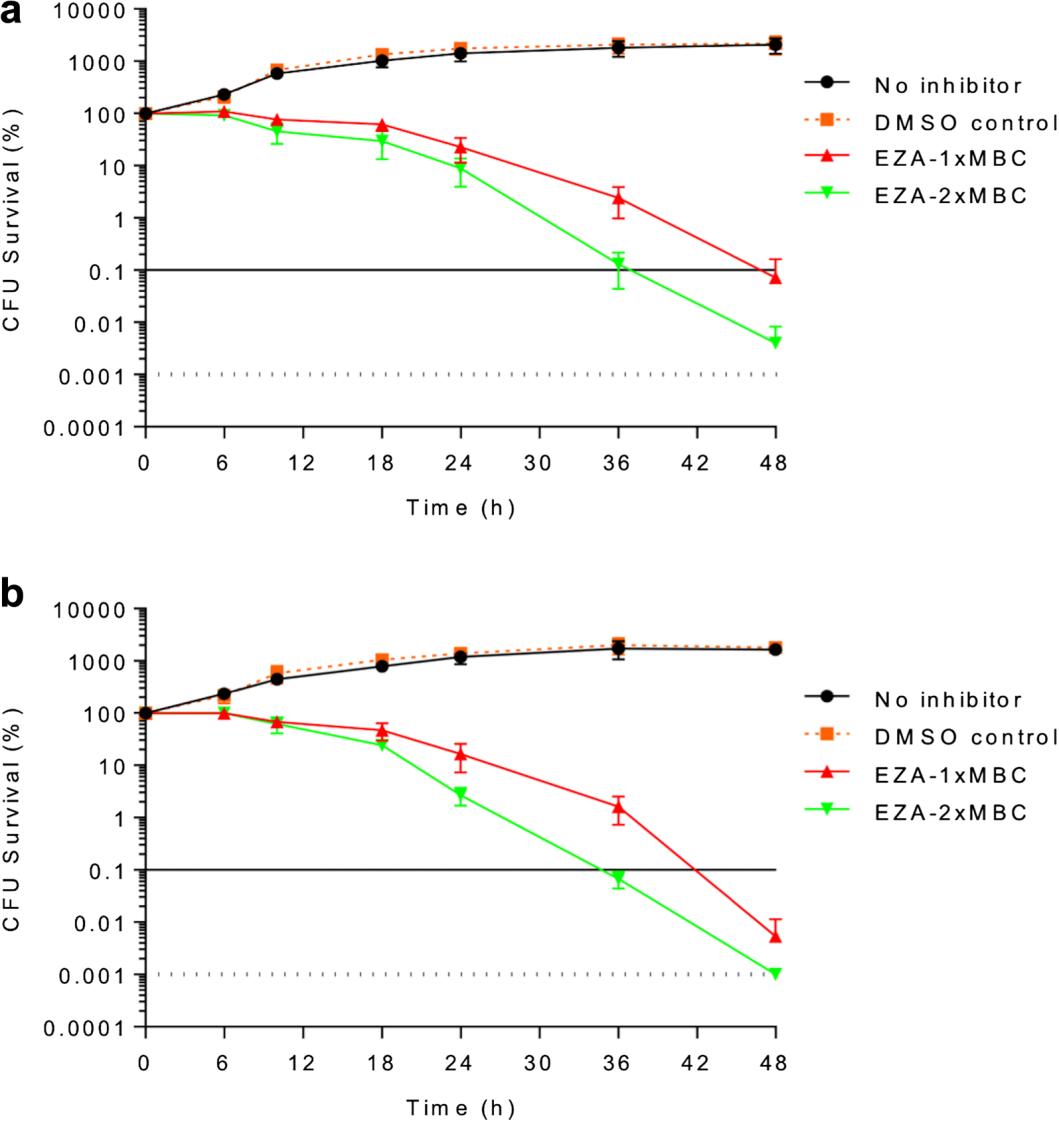
Time and dose dependency of the antimicrobial action of EZA on strains 26695 (**a**) and SS1 (**b**). Bactericidal kinetics is shown for 1 × MBC and 2 × MBC of EZA. The horizontal dashed line represents the limit of detection (100 cells) and the horizontal solid line corresponds to 99.9% cell death. Error bars represent the standard error of the mean for three independent biological replicates

It has been previously shown that EZA displays antimicrobial activity against *H. pylori* strains SS1 (MIC = 0.2 mM, MBC = 0.4 mM) and 26695 (MIC = 0.3 mM, MBC = 0.5 mM) [11]. To enhance our understanding of its bactericidal properties, we assessed time-dependent killing kinetics at concentrations corresponding to 1 × MBC and 2 × MBC. For both strains, 99.9% of cells were killed following a 36-h exposure to 2 × MBC of EZA (Fig. 1a, b). When 1 × MBC of EZA was used, 42 h and 48 h were required to kill 99.9% of SS1 (Fig. 1b) and 26695 cells (Fig. 1a), respectively. The DMSO solvent (1% control) had no detectable effect on cell viability under the experimental conditions (Figs. 1a, b). This analysis has also shown that bactericidal activity of EZA against both strains is concentration-dependent.

### Isolation and characterization of EZA-resistant mutants of H. pylori strains SS1 and 26695

Mutants of *H. pylori* strains SS1 and 26695 with significantly reduced susceptibility to EZA were identified following selection by serial passages in liquid culture in the presence of sub-lethal concentrations of the inhibitor. The EZA-resistant SS1 strain (hereafter referred to as SS1 EZA) had more than a tenfold increase in the MIC (MIC > 2 mM) from its wild-type (WT) value (0.2 mM). Similarly, the EZA-resistant strain 26695 (hereafter referred to as 26695 EZA) had an MIC > 3 mM, which is > 10 × WT MIC. The resistance phenotype remained stable in the absence of selection (monitored for 2 weeks).

In view of the potential use of EZA as a lead for antibacterial drug discovery, it is important to know if resistance to it develops readily. Estimation of the rate at which spontaneous mutations conferring resistance to EZA occur was performed using a single selection step with EZA at a concentration corresponding to 10 × MIC. For both strains, the frequency of spontaneous resistant mutants was < 10^−8^. For comparison, we have also determined the frequency of spontaneous mutations leading to resistance to rifampicin, obtaining values of 10^−6^ for SS1 and 5 × 10^−6^ for 26695. As the frequency of spontaneous acquisition of resistance to EZA was significantly lower than that for rifampicin, or those previously reported for other commercial antibiotics, such as metronidazole or tetracycline (10^−5^–10^−6^) [16], it is evident that resistance to EZA in *H. pylori* strains SS1 and 26695 does not develop easily.

### Identification of genetic determinants associated with resistance to EZA in strains SS1 and 26695

To explore the genetic mechanisms that underpin resistance to EZA in *H. pylori* strains SS1 and 26695, we first sequenced and compared the full genomes of the EZA-resistant isolates and their respective WT parental strains. We found no evidence of significant genome re-organization (i.e. no large insertions, deletions or recombinations). All observed differences were single or double nucleotide substitutions, or single or double nucleotide insertions/deletions (listed in Additional file 1: Table S1 and Additional file 2: Table S2). SS1 EZA and 26695 EZA harbored mutations in 9 and 14 genes, respectively. None of these mutations were located in the genes encoding HpαCA or HpβCA, excluding the possibility that modifications of these two known targets of EZA resulted in the resistance phenotype. To eliminate random mutations with no link to EZA resistance, the mutant chromosomal DNA was isolated and transformed back into the WT background, followed by selection of resistant transformants on plates containing 10 × MIC of EZA. Sequencing of all the candidate genes in the resultant EZA-resistant recombinants (hereafter referred to as SS1 TF and 26695 TF) revealed that SS1 TF retained mutations in only 3 genes (Table 1), whereas 26695 TF retained mutations in 6 genes (Table 2). No mutations were common to both resistant recombinants. SS1 TF had a frameshift mutation in the gene encoding the flagellar protein export apparatus component FliO, a Val464Ala substitution in endoribonuclease RNase Y, and an Ala302Ser substitution in the O-antigen flippase Wzk. 26695 TF had mutations resulting in amino-acid substitutions in RNA polymerase subunit α (Glu290Lys), ATP synthase subunit δ (Glu171Lys), undecaprenyl pyrophosphate synthase UppS (Asn131Asp), flavin prenyltransferase UbiX (Gly60Ala) and in a hypothetical protein encoded by HP_0781 (Ala92Val, Asp102Glu, and Thr201Ala).

**Table 1:** Nucleotide changes in *H. pylori* SS1 TF generated by transformation of SS1 with SS1 EZA genomic DNA

Position^a^	Type	Reference	Allele	Locus tag	Amino acid substitution	Gene product
592353	Substitution	T	C	HPYLSS1_00577	Val464Ala	Ribonuclease Y
1013480	Deletion	A	–	HPYLSS1_00787	Lys212fs^b^	Flagellar protein export apparatus component FliO
1235634	Substitution	G	T	HPYLSS1_01157	Ala302Ser	O-antigen flippase Wzk

^a^Positions of nucleotides are specified with reference to the published *H. pylori* SS1 genome (GenBank ID: CP009259 [17])

^b^fs, frameshift mutation

**Table 2:** Nucleotide changes in *H. pylori* 26695 TF generated by transformation of 26695 with 26695 EZA genomic DNA

Position^a^	Type	Reference	Allele	Locus tag	Amino acid substitution	Gene product
1367889	Substitution	C	T	HP_1293	Glu290Lys	RNA polymerase subunit α
1197822	Substitution	C	T	HP_1134	**–**	ATP synthase subunit α
1198143	Deletion	TC	–	–	–	Ribosome-binding site for HP_1134
1198189	Substitution	T	C	HP_1135	Glu171Lys	ATP synthase subunit δ
1296698	Substitution	T	C	HP_1221	Asn131Asp	UPP pyrophosphate synthase
1548534	Substitution	A	C	HP_1476	Gly60Ala	Flavin prenyltransferase UbiX
835376	Substitution	T	C	HP_0781	Ala92Val	Hypothetical protein
835407	Substitution	A	C		Asp102Glu	
835710	Substitution	G	A		Thr201Ala	

^a^Nucleotide positions are indicated with reference to the published *H. pylori* 26695 genome (GenBank ID: AE000511 [18])

## Discussion

*Helicobacter pylori* is the primary cause of chronic gastritis, peptic ulcers, mucosa-associated lymphoid tissue lymphoma and gastric adenocarcinoma. With the rise of resistance to conventional antibiotics, *H. pylori* has become a high priority pathogen for development of new antimicrobials. It has been known for some time that there is a safe, FDA-approved drug, EZA, originally designed to treat non-infectious human illnesses (e.g. glaucoma), that shows antimicrobial activity against *H. pylori*. The potential of this compound to be re-purposed, or to be used as a lead compound for development of a novel anti-*H. pylori* drug warrants studies of its mechanisms of antibacterial action and pathways to resistance.

To date, EZA activity and resistance have been investigated for only one *H. pylori* strain, P12 [11]. In this study, we have performed a susceptibility and resistance study with *H. pylori* strains SS1 and 26695. Analysis of the commonalities between the resistance determinants in different *H. pylori* strains provides important insights into the mechanism of anti-*H. pylori* activity of EZA.

The study was undertaken at neutral pH, as this approximates the conditions under which *H. pylori* persists in the mucous layer adjacent to the gastric epithelium. As with P12, EZA displayed bactericidal activity against strains SS1 and 26695 in a time- and concentration-dependent manner. Similar to P12, spontaneous resistant mutants of strains SS1 and 26695 isolated at neutral pH had changes in several genes, none of which encoded the known targets of EZA, HpαCA or HpβCA. The complex genotype associated with *H. pylori* resistance to EZA therefore indicates that EZA targets multiple cellular proteins and systems other than α- and β-carbonic anhydrases. Simultaneous changes to several different functions are required for resistance to emerge, which is consistent with our observation that, as with P12, resistance to EZA in strains SS1 and 26695 does not develop easily (rate of spontaneous resistance acquisition < 10^−8^).

Analysis of the resistance-conferring genetic changes, that were identified by backcrossing the mutations into the parent strain, allowed us to develop hypotheses about resistance mechanisms and discuss the implications for the mechanism of antimicrobial activity of EZA. Although there were no common mutations conferring resistance to EZA in *H. pylori* strains P12, SS1 and 26695, all three mutant strains contained alterations in genes encoding proteins involved in cell wall synthesis and in control of gene expression. In common with the resistant P12 mutant, 26695 TF contained a mutation in the gene encoding undecaprenyl pyrophosphate synthase (UppS), albeit at a different site (Asn131Asp in 26695, Glu173Lys in P12). UppS plays an essential role in the cell wall biosynthesis. It generates undecaprenyl pyrophosphate (Upp) on the cytoplasmic side of the inner membrane [19]. Upp is then dephosphorylated by an, as yet, unidentified pyrophosphatase to form undecaprenyl phosphate—a universal glycan lipid carrier which is used to translocate building blocks of peptidoglycan and lipopolysaccharide (LPS) O-antigen across the inner membrane into the periplasm [20]. The observation that the essential *uppS* gene is mutated in two out of the three resistant strains suggests that EZA directly targets this enzyme in killing *H. pylori*. Indeed, other sulfonamides (BTB06061 and HTS04781) have been previously shown to inhibit *H. pylori* UppS with IC_50_ in the micromolar range [21]. Furthermore, out of the two mutations found in *uppS*, one (Glu173Lys) changes a residue in the enzyme active site [11], which supports the notion that EZA likely acts as a competitive inhibitor of UppS by mimicking its natural substrate, transition state or product.

Although the *uppS* gene was not altered in the SS1 mutant resistant to EZA, we have found a mutation in the *wzk* gene encoding the O-antigen flippase, a protein in the same biosynthetic pathway. In the course of the LPS synthesis, the O-antigen component of the LPS is fully assembled on the glycan lipid carrier produced by UppS prior to being translocated (flipped) onto the periplasmic side of the inner membrane by Wzk [22]. By mimicking the glycan lipid carrier, EZA may be able to inhibit multiple proteins in the Upp-related pathways (in this instance, both UppS and Wzk). Cells appear to acquire resistance by altering the proteins in the O-antigen biosynthesis pathway to remove favorable interactions with the inhibitor. Inhibitory activities of EZA on UppS and WzK are thought to lead to cell death in WT strains via different mechanisms. Inactivating UppS kills cells by blocking the cell wall synthesis [19, 20]. In contrast, *wzk* mutants of *H. pylori* are viable [20], but they lack O-antigen, and hence, the protective outer hydrophilic layer that, in WT cells, repels hydrophobic compounds such as EZA. Wzk inhibition by EZA would therefore increase cell wall permeability of this compound, aiding its access to other, intracellular targets.

Changes in the genes encoding proteins that control gene expression likely affect the global regulation of metabolic enzymes, altering cell physiology in a way that aids resistance. In strain P12, spontaneous mutants resistant to EZA possessed a single amino-acid substitution in the transcription termination factor NusA [11]. This study revealed that EZA-resistant mutants of strains SS1 and 26695 also contained mutations that likely affect control of transcription, albeit in different genes. The SS1 mutant had a single amino-acid substitution (Val464Ala) in endoribonuclease RNase Y—an enzyme that regulates the expression of hundreds of genes via processing, stabilization or degradation of their mRNA [23]. In addition, it had a frameshift mutation in the *fliO* gene, which was shown to be required for transcription of many RpoN (σ54)- and FliA (σ28)-dependent genes in *H. pylori* [24]. Finally, in the EZA-resistant mutant of strain 26695, we found a Glu290Lys substitution in the conserved EEE motif of the C-terminal domain of the RNA polymerase subunit α that interacts with NikR, a transcription factor that regulates multiple genes [25].

Furthermore, the EZA resistant isolate of strain 26695 contains a mutation in the gene from the metabolic pathway that is connected to the carbonic anhydrase-catalyzed reaction: flavin prenyltransferase UbiX. The UbiD-UbiX decarboxylase system is involved in the ubiquinone biosynthesis pathway; it catalyzes decarboxylation of 3-octaprenyl-4-hydroxybenzoic acid [26]. As carbonic anhydrases equilibrate CO_2_ and bicarbonate, the levels of their activity probably affect the rate of the UbiD-UbiX-catalysed reaction, hence influencing the production of ubiquinone. It is therefore thought that the conservative substitution Gly60Ala in UbiX may be compensating for the altered levels of CO_2_ due to the inhibition of HpαCA or HpβCA by EZA.

In conclusion, the comparative analysis of the genotypes of EZA-resistant mutants of three different *H. pylori* strains revealed that strain-specific pathways to resistance exist, strengthening the notion that EZA targets multiple systems. Indeed, the rate of spontaneous resistance acquisition in *H. pylori* was determined to be < 10^−8^, demonstrating that resistance does not develop easily. Resistant isolates of all three *H. pylori* strains contained mutations in genes encoding proteins involved in cell wall synthesis and in control of gene expression. Our study has highlighted the O-antigen synthesis pathway, and UppS in particular, as one of the key putative targets of EZA. Although no mutations were found in the genes for the known targets of EZA—HpαCA and HpβCA—there is a strong possibility that inhibition of these enzymes contributes to the antimicrobial action of this drug, as a mutation in the gene (*ubiX*) from the metabolic pathway connected to the carbonic anhydrase-catalyzed reaction has also been found. The new insights obtained in this study provide a useful foundation for future biochemical and genetic studies to explore the potential for the development of EZA into a novel anti-*H. pylori* drug.

## Methods

### *Helicobacter pylori* strains, reagents, media, and growth conditions

*Helicobacter pylori* strains SS1 [27] and 26695 [28] were used for this study. *H. pylori* was cultured on Columbia blood agar (Oxoid) with 5% (*v/v*) defibrinated horse blood (Australian Ethical Biologicals), 10 µg/ml vancomycin, 5 µg/ml cefsulodin, 2.5 U/ml polymyxin B, 5 µg/ml trimethroprim, and 8 µg/ml amphotericin B (all antibiotics from Sigma-Aldrich). Liquid cultures were grown with shaking at 120 rpm in Brucella broth (Becton–Dickinson) with 10 µg/ml vancomycin and 10% (*v/v*) foetal bovine serum (FBS). Cultures were incubated at 37 °C under microaerophilic conditions generated using the CampyGen (Oxoid) system. EZA (Sigma-Aldrich) was dissolved in dimethyl sulfoxide (DMSO, Sigma-Aldrich) at a stock concentration of 100 mM, and stored at − 20 °C. All antibiotics were dissolved and diluted according to Clinical and Laboratory Standard Institute (CLSI) guidelines [29].

### Evaluation of bactericidal kinetics of EZA in *H. pylori* strains SS1 and 26695

The minimum inhibitory concentrations (MICs) and minimum bactericidal concentrations (MBCs) of EZA for *H. pylori* strains SS1 and 26695 were determined as previously described [11]. To assess bactericidal kinetics of EZA, bacterial cells were grown in liquid medium to an optical density at 600 nm (OD_600_) of 0.4–0.6, pelleted, washed, resuspended in antibiotic-free medium to an OD_600_ of 0.05 (equivalent to 10^4^ to 10^5^ cells/ml), and aliquoted in 1 mL volumes supplemented with EZA at a concentration of 1 × MBC or 2 × MBC. A control culture was supplemented with 1% DMSO to ascertain that the DMSO solvent did not affect the bacterial viability. The cells were allowed to continue growing, and the viable cells were enumerated at time points 0, 6, 12, 18, 24, 36, and 48 h by plating the 10^−10^, 10^−9^, and 10^−8^ dilutions of the cultures on non-selective horse blood agar (HBA) plates and counting the colonies.

### Isolation and characterization of *H. pylori* SS1 and 26695 mutants resistant to EZA

EZA-resistant mutants of *H. pylori* strains SS1 and 26695 (referred to as SS1 EZA and 26695 EZA) were obtained by iterative selection for progressive resistance to 0.25 × MIC, 0.5 × MIC and then 10 × MIC, using the approach previously described for *H. pylori* strain P12 [11]. The approximate frequency of pre-existing spontaneous mutations allowing growth at 10 × MIC of EZA was determined using single step selection on plates inoculated with 2 × 10^6^, 2 × 10^7^ or 2 × 10^8^ CFU, with EZA included in the solid medium. Single-step selection on plates containing 10 μg/mL rifampicin were used as a control. The mutation frequency was calculated as $\frac{\text{average CFU counted on plates supplemented with EZA}}{\text{CFU in the inoculum}}$. To ascertain the stability of the resistant phenotype, SS1 EZA and 26695 EZA mutants were passaged 5 times on EZA-free plates. The MIC and MBC values for EZA in the passaged isolates did not differ significantly from those of the original EZA-resistant strains.

### Elimination of background mutations by backcrossing to wild type

Mutations responsible for the EZA-resistance phenotype were separated from spontaneous background mutations by isolating the genomic DNA of SS1 EZA and 26695 EZA and transforming it into the WT SS1 and 26695 strains, respectively. Genomic DNA extracted from WT SS1 and 26695 strains, and buffer, were used as controls. Resulting transformant colonies resistant to EZA (hereafter referred to as SS1 TF and 26695 TF), were selected on Colombia blood agar plates containing 10 × MIC of EZA.

### Genome sequencing and analysis

Genomic DNA was isolated from both WT and EZA-resistant SS1 and 26695 strains with the GenElute Bacterial Genomic DNA kit (Sigma-Aldrich). Libraries were prepared using the NexteraXT (Illumina) and quantified using the Qubit DNA HS kit (Invitrogen). Whole genome sequencing (WGS) was performed at the Micromon High-Throughput Sequencing Facility (Monash University) using the Illumina MiSeq platform with a paired end configuration and a 150 bp average read length.

Analysis of the WGS data was carried out using CLC Genomics Workbench v. 7.0.3 (Qiagen). Reads were aligned to the publicly available reference genomes of *H. pylori* SS1 and 26695 (NCBI accession numbers CP009259 and AE000511.1). Genomic differences between the WT parental strain and the EZA-resistant mutant were identified using the Probalistic Variant Detection and the Quality Based Variant Detection analysis tools in CLC Genomics Workbench, and subsequently confirmed by Sanger sequencing at Micromon (see below).

### Sanger sequencing

The genes of interest were amplified by PCR using genomic DNA as a template, purified using the Wizard SV gel and PCR clean up kit (Promega), and sequenced using the Sanger method. Sequence alignment and visualization were performed using BioEdit v 7.0.5 (http://www.mbio.ncsu.edu/bioedit/bioedit.html).

## Supplementary information

**Additional file 1: Table S1.** Nucleotide changes in the *H. pylori* SS1 mutant resistant to EZA.

**Additional file 2: Table S2.** Nucleotide changes in the *H. pylori* 26695 mutant resistant to EZA.

## Data Availability

All data generated or analysed during this study are included in this published article and its additional information files. Strains are available from the corresponding author on request.
