# Supplementary material for: Antibacterial activity of ethoxzolamide against Helicobacter pylori strains SS1 and 26695
Source: Gut Pathog. 2020 Apr 15;12:20. doi: 10.1186/s13099-020-00358-5 (PMC7158114; doi:10.1186/s13099-020-00358-5)
Supplement: Supplementary file 1 — Additional file 1: Table S1. Nucleotide changes in the H. pylori SS1 mutant resistant to EZA. [file 13099_2020_358_MOESM1_ESM.pdf]

Table S1. Nucleotide changes in the *H. pylori* SS1 mutant resistant to EZA.

Position[a]	Type	Reference	Allele	Locus tag	Amino acid substitution	Gene product
***Helicobacter pylori* SS1 EZA resistant mutant**						
**Gene class: transporter**						
309374	Deletion	G	-	HPYLSS1_00289	Ala250fs[b]	dipeptide transport system permease protein DppC
1013480	Deletion	A	-	HPYLSS1_00787	Lys212fs	flagellar protein export apparatus component FliO
**Gene class: regulation**						
592353	Substitution	T	C	HPYLSS1_00577	Val464Ala	ribonuclease Y
**Gene class: translation**						
1169965	Substitution	C	A	HPYLSS1_01099	Gly311Val	valyl-tRNA synthetase
**Gene class: cell wall synthesis**						
1235634	Substitution	G	T	HPYLSS1_01157	Ala302Ser	O-antigen flippase Wzk
1415084	Deletion	GA	-	HPYLSS1_01343	Glu263fs	phosphoethanolamine transferase
**Gene class: biological function unknown**						
1004529	Insertion	-	TG	HPYLSS1_00797	His2fs	haloacid dehalogenase-like hydrolase
1030442	Substitution	G	T	HPYLSS1_00769	Gly45Trp	hypothetical protein
1306813	Substitution	A	C	HPYLSS1_01230	-	hypothetical protein

[a]Position of nucleotides are specified with reference to the published *H. pylori* SS1 genome (GenBank ID: CP009259)

[b]fs, frameshift mutation
