# Supplementary material for: Antibacterial activity of ethoxzolamide against Helicobacter pylori strains SS1 and 26695
Source: Gut Pathog. 2020 Apr 15;12:20. doi: 10.1186/s13099-020-00358-5 (PMC7158114; doi:10.1186/s13099-020-00358-5)
Supplement: Supplementary file 2 — Additional file 2: Table S2. Nucleotide changes in the H. pylori 26695 mutant resistant to EZA. [file 13099_2020_358_MOESM2_ESM.pdf]

Table S2. Nucleotide changes in the *H. pylori* 26695 mutant resistant to EZA.

Position[a]	Type	Reference	Allele	Locus tag	Amino acid substitution	Gene product
***Helicobacter pylori* 26695 EZA resistant mutant**						
**Gene class: membrane proteins and transporters**						
57000	Substitution	C	A	HP_0055	Val239Phe	sodium/proline symporter
524749	Insertion	-	T	HP_0498	Ser223fs[b]	sodium-dependent transporter
646044	Substitution	C	T	HP_0607	Ala862Val	efflux RND transporter permease subunit
1248093	Substitution	A	C	HP_1180	Leu360Trp	nucleoside transporter
**Gene class: translation**						
1281494	Substitution	G	A	HP_1205	Pro129Ser	translation elongation factor Tu
**Gene class: transcription**						
1367889	Substitution	C	T	HP_1293	Glu290Lys	RNA polymerase subunit α
94302	Substitution	C	A	HP_0088	Glu222Asp	RNA polymerase σ-factor
**Gene class: ATP synthase**						
1197822	Substitution	C	T	HP_1134	-	ATP synthase subunit α
1197875	Substitution	C	T		Gly88Ser	
1198143	Deletion	TC	-	-	-	ribosome-binding site for HP_1134
1198189	Substitution	T	C	HP_1135	Glu171Lys	ATP synthase subunit δ
**Gene class: cell wall synthesis**						
1296698	Substitution	T	C	HP_1221	Asn131Asp	UPP pyrophosphate synthase
**Gene class: metabolic**						
749176	Insertion	-	A	HP_0696	Lys408fs	acetone carboxylase subunit α
1548534	Substitution	A	C	HP_1476	Gly60Ala	flavin prenyltransferase UbiX
1548564	Substitution	C	T		Ala129Thr	
1548769	Substitution	AC	CG		Ser139Ala	
**Gene class: biological function unknown**						
597699	Deletion	TC	-	HP_0565	Glu81fs	hypothetical protein
835376	Substitution	T	C	HP_0781	Ala92Val	hypothetical protein
835407	Substitution	A	C		Asp102Glu	
835710	Substitution	G	A		Thr201Ala	

[a]Nucleotide positioned are indicated with reference to the published *H. pylori* 26695 genome (GenBank ID: AE000511).

[b]fs, frameshift mutation
